# Can T1W and T2W Dixon Sequences Replace the Standard MRI Protocol in Diagnosing Sacroiliitis?

**DOI:** 10.5334/jbsr.3658

**Published:** 2024-12-12

**Authors:** Nur Betül Karatoprak, Zeynep Maraş Özdemir, Sinan Karatoprak, Ayşegül Sağır Kahraman, Leyla Karaca, Servet Yolbaş

**Affiliations:** 1 Department of Radiology, Kayseri City Training and Research Hospital, Turkey; 2 Faculty of Medicine, Department of Radiology, İnönü University, Turkey; 3 Faculty of Medicine, Departments of Internal Medicine, İnönü University, Turkey

**Keywords:** Sacroiliitis, magnetic resonance imaging, chemical shift imaging

## Abstract

*Objectives:* This study aims to assess the performances of T1‑weighted (T1W) and T2‑weighted (T2W) Dixon sequences as replacements for the standard magnetic resonance imaging (MRI) protocol for diagnosing active and chronic sacroiliitis.

*Materials/Methods:* This single‑centre, prospective study included 107 patients who underwent 3 Tesla MRIs. The patients with inflammatory low‑back pain (aged 18–50 years) were included. The exclusion criteria included pregnancy, pelvic infection/malignancy history, pelvic metal implants or foreign body artefacts. The imaging protocol comprised standard T1W and T2W fat‑saturated (T2W‑FS) sequences and T1W–T2W Dixon sequences. Active sacroiliitis signs were assessed by comparing T2W‑FS images with T2W Dixon water‑only (WO) images. Chronic sacroiliitis signs were evaluated by comparing the standard T1W sequence with T1W–T2W Dixon fat‑only (FO), in‑phase (IP) and out‑of‑phase (OP) images. The quantitative analysis involved calculating signal‑to‑noise ratios (SNRs) and contrast‑to‑noise ratios (CNRs) for bone marrow edema (BME) and periarticular fat deposition (PFD). Descriptive statistics, correlation, diagnostic performance tests and interobserver reliability tests were performed in the qualitative analysis.

*Results:* There were no statistically significant differences in BME detection between the T2W‑FS and T2W Dixon‑WO images. T2W Dixon exhibited significantly greater SNRs–CNRs than did the standard protocol for BME and periarticular fat deposition assessments. T1W–T2W Dixon imaging demonstrated sufficiently high diagnostic performance for detecting erosions, periarticular fat deposition and ankylosis compared with the standard protocol.

*Conclusions:* The T2W Dixon sequence has the potential to replace the standard protocol, which would reduce acquisition time. However, we do not recommend the use of the T1W Dixon sequence in routine practice, since standard T1W images provide similar or superior results to T1W Dixon images.

## Introduction

Sacroiliitis, inflammation of the sacroiliac joint (SIJ), is the most important cause of inflammatory low back pain and is often associated with axial spondyloarthropathies [1]. Early diagnosis and treatment are important for preventing or delaying irreversible disease [2, 3].

The best imaging method for diagnosing sacroiliitis is magnetic resonance imaging (MRI) because it reveals both signs of active inflammation and chronic structural damage comparable with those of computed tomography [4, 5]. In general, sacroiliac MRI protocol includes T1‑weighted (T1W) sequences to show signs of chronic sacroiliitis and T2 weighted fat saturated (T2W‑FS) sequences to show active inflammation [6].

Dixon imaging provides more homogeneous fat suppression than other fat suppression methods. It provides four different images, ‘water only (WO)’, ‘fat only (FO)’, ‘in‑phase (IP)’ and ‘out‑of‑phase (OP)’, in only one acquisition. Although there are several studies in the literature evaluating the use of the Dixon sequence in the diagnosis of active and chronic sacroiliitis thus far, they have different results, especially in showing chronic sacroiliitis findings, and none has used the T1W Dixon sequence [7–11].

In this study, we aim to determine the performance of T1W and T2W Dixon sequences in the diagnosis of active and chronic sacroiliitis by comparing those imaging data with the imaging data obtained with standard sacroiliac MR imaging.

## Material and Methods

We obtained approval from the institutional review board and informed consent from all participants. This prospective single‑centre study was performed at the authors’ institution over a 3‑month period.

### Study population

The patients who underwent rheumatologist‑ordered MRI for suspicion of sacroiliitis were evaluated for the study. The inclusion criteria were the presence of inflammatory low back pain complaints and aged 18–50 years. Patients who were pregnant, had a history of pelvic infection, malignancy, or had pelvic metal implants or foreign body artifacts were excluded from the study.

### MRI protocols

All MRIs were performed on a 3‑Tesla machine (Magnetom Skyra, Siemens, Erlangen, Germany) with the patient in the supine position using a combination of 32‑channel flexible posterior and 18‑channel anterior coil.

Standard protocol for sacroiliac imaging at our centre included coronal oblique and axial oblique fat‑saturated turbo spin‑echo T2W and coronal oblique turbo spin‑echo T1W sequences. Contrast‑enhanced sequences were not obtained based on The Assessment of Spondyloarthritis International Society (ASAS) guidelines [6, 12].

T1W–T2W multipoint Dixon sequences with four images were added to the standard protocol, which was performed with similar planes and parameters (Table 1).

**Table 1:** Magnetic resonance imaging acquisition parameters for the sacroiliac joints. TR: time to repeat, TE: time to echo.

PARAMETERS	SECTION THICKNESS (MM)	FIELD OF VIEW (MM × MM)	MATRIX	TR/TE	ACQUISITION TIME (MIN:S)
T1W	3	270	265 × 448	402/22	2:44
T2W‑FS	3	270	216 × 320	3220/72	1:41
T1W‑Dixon	3	250	195 × 320	607/10	2:11
T2W‑Dixon	3	270	202 × 320	4620/81	3:11

### Image analysis

All images were evaluated by two radiologists with 3 and 7 years of MRI experience (N.B.K. and S.K.) who were blinded to the clinical data and final diagnosis of the patients. In accordance with the standard MRI sequences (T1W and T2W‑FS sequences), T1W Dixon and T2W Dixon sequences were evaluated independently and separately by the same two radiologists at three different reading sessions with 15‑day intervals. After these evaluations, these two radiologists made a consensus on the findings to be used in the quantitative analysis.

In patient groups in which two radiologists agreed on the presence of findings, the quantitative evaluation was performed by the first radiologist (N.B.K.) 4 weeks after the qualitative evaluation. Patients with disagreements were not included in the quantitative analysis.

### Qualitative assessments

According to the ASAS/OMERACT criteria, active and chronic sacroiliitis findings were evaluated [13].

In the diagnosis of active sacroiliitis, bone marrow edema (BME) from coronal T2W FS and coronal T2 Dixon WO images was examined.

MRI findings of synovitis, enthesitis and capsulitis were ignored because these findings are not sufficient to diagnose active sacroiliitis in the absence of BME according to ASAS criteria.

In the diagnosis of chronic sacroiliitis, the presence of erosion, subchondral sclerosis, periarticular fat deposition (PFD), backfill and ankylosis were examined. Coronal T1W, T1W–T2W Dixon FO, IP and OP images were evaluated.

### Quantitative assessments

Quantitative evaluation of BME and PFD lesions was performed. Measurements were made from the most prominent lesion, normal bone marrow (sacral interforaminal region) and air (reference sample) with a 5‑mm‑diameter region of interest.

BME was measured on T2W‑FS images and T2W Dixon WO images. PFD was measured on standard T1W images and T1W–T2W Dixon FO, IP and OP images. Signal‑to‑noise ratios (SNRs) and contrast‑to‑noise ratios (CNRs) were calculated.

### Statistical analysis

Continuous data normality was assessed using analytical methods (Kolmogorov–Smirnov/Shapiro–Wilk tests), along with consideration of skewness and kurtosis coefficients. Nonparametric tests were employed due to the dataset’s lack of normal distribution and insufficient sample size for parametric tests.

For two‑sample analysis, the Mann‒Whitney *U* test was used. The Kruskal‒Wallis *H* test was applied to analyse more than two independent samples. Wilcoxon conjugate‑pair and McNemar tests were utilized for two related samples. Friedman and Cochran’s *Q* tests were employed for the analysis of more than two related samples.

To determine the diagnostic performance of T1W and T2W Dixon imaging, the standard MRI protocol was accepted as the gold standard imaging method and specificity, sensitivity, positive predictive value, negative predictive value and accuracy were calculated. Interobserver agreements were made with Cohen kappa analysis. Kappa values were categorized as follows: up to 0.20 for slight agreement, 0.21–0.40 for fair agreement, 0.41–0.60 for moderate agreement, 0.61–0.80 for substantial agreement, 0.81–0.99 for almost perfect agreement and 1 for perfect agreement [14].

We used statistical software (SPSS, version 22.0, Chicago, Illinois, USA) for data analysis, and *P* values < 0.05 were considered to indicate statistical significance.

## Results

### Study population

A total of 214 sacroiliac joints of one hundred and seven patients were included in this study (73 females and 34 males, mean age 36 years) (Table 2).

**Table 2:** Characteristics of the our study sample according to standard MRI protocol findings.

STANDARD MRI PROTOCOL FINDINGS	NUMBER OF CASES	AGE RANGE (YEARS)	MEAN AGE (YEARS)	GENDER (FEMALE/MALE)
Positive active sacroiliitis findings	6	35–45	39.33	6/0
Positive chronic sacroiliitis findings	14	18–47	33.78	7/7
Active on chronic sacroiliitis findings	29	21–50	36.06	22/7
No active or chronic sacroiliitis findings	58	18–48	36.37	38/20
Total	107	18–50	36.12	73/34

### Qualitative assessments

The results of the qualitative analysis of the sacroiliac joints with positive findings on MRI are summarized in Table 3.

**Table 3:** The number of sacroiliac joints with positive findings on MRI sequences.

MRI FINDINGS	T2W‑FS	T1W	T1W DIXON FO	T1W DIXON IP	T1W DIXON OP	T2W DIXON WO	T2W DIXON FO	T2W DIXON IP	T2W DIXON OP
BME	56	–				57			
Erosion		87	85	85	71		82	80	68
Subchondral sclerosis		43	21	22	5		22	20	15
PFD		23	21	21	20		21	21	21
Ankylosis		5	4	4	4		4	4	4
Backfill		7	6	4	0		3	5	5

**Figure 1:**
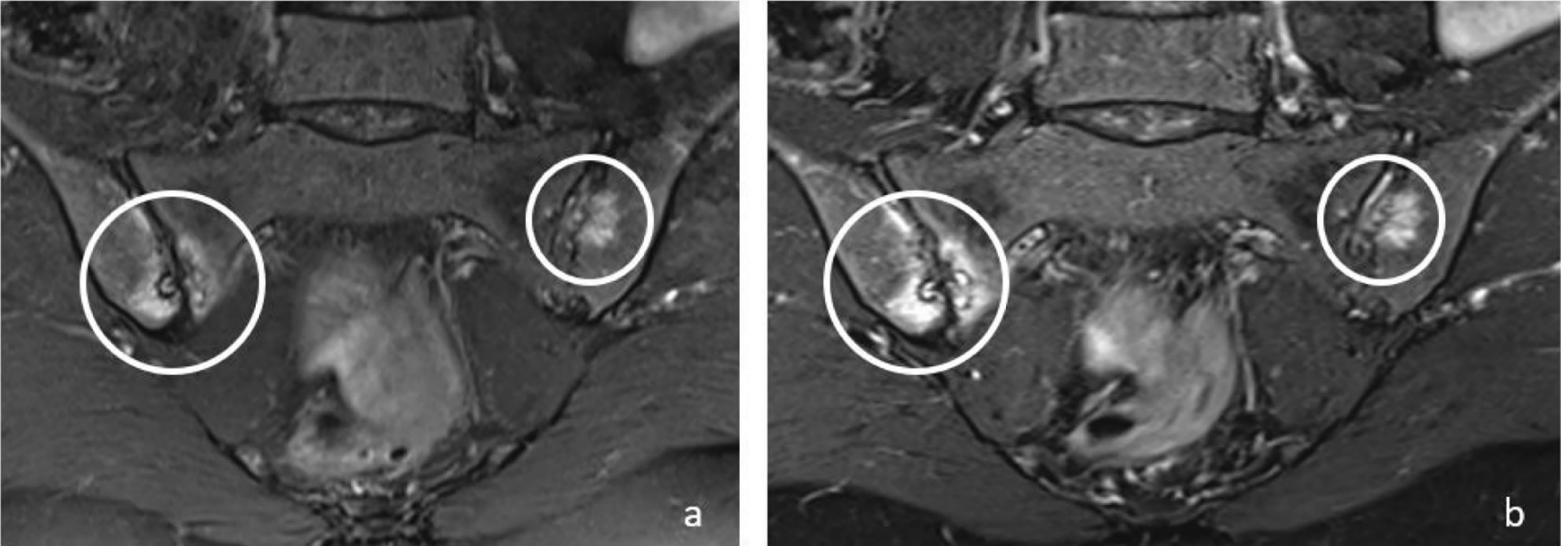
Coronal T2W‑FS **(a)** and T2W Dixon WO **(b)** images show bone marrow edema (circles) in both sacroiliac joints on the right sacral and iliac side and on the left iliac side in a 48‑year‑old female patient. Bone marrow edema signals are brighter on T2W Dixon WO images **(b)**.

In the evaluation of active sacroiliitis, there was no statistically significant difference between standard FS‑T2W and T2W Dixon WO images in detection of BME (*P* > 0.05) (Figure 1).

**Figure 2:**
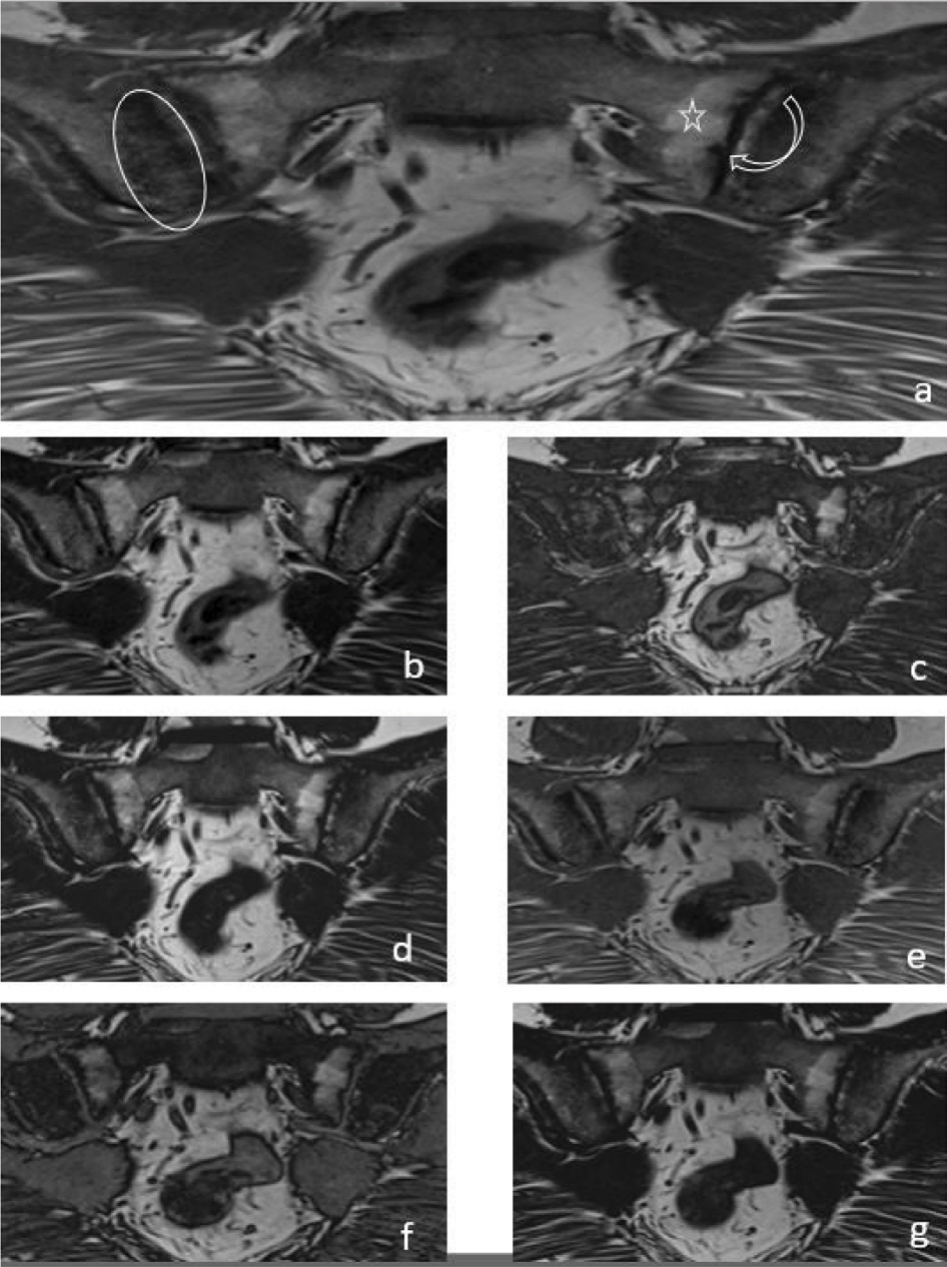
A 33‑year‑old female patient with iliac side sclerosis (ovoid circle), sacral side periarticular fat deposits (star) and iliac‑sacral facial erosions (curved arrow) in both sacroiliac joints marked on a T1W images **(a)**. These findings can also be seen in T2W Dixon IP **(b)**, T2W Dixon OP **(c)**, T2W Dixon FO **(d)**, T1W Dixon IP **(e)**, T1W Dixon OP **(f)** and T1W Dixon FO **(g)** images.

**Figure 3:**
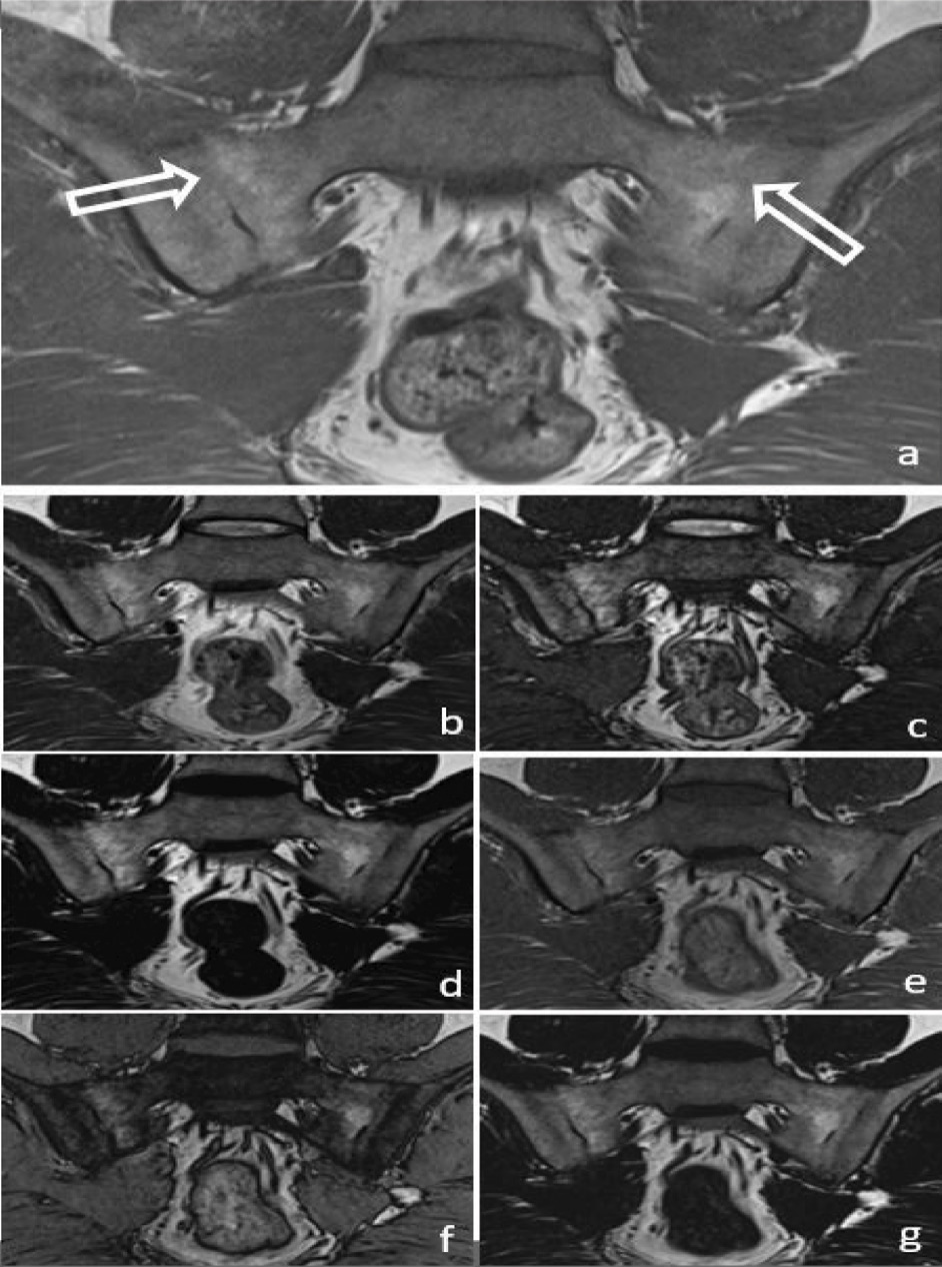
A 34‑year‑old male patient with ankylosis (arrow) in both sacroiliac joints on a T1W image **(a)** (arrows). Ankylosis can be easily seen in the T2W Dixon IP **(b)**, T2W Dixon OP **(c)**, T2W Dixon FO **(d)**, T1W Dixon IP **(e)**, T1W Dixon OP **(f)** and T1W Dixon FO **(g)** images shown in the figure.

In the evaluation of chronic sacroiliitis, there was no significant difference in the detection of PFD (*P* = 0.08) (Figure 2) or ankylosis (*P* = 0.42) (Figure 3) among standard T1W, T1W–T2W Dixon (FO, IP and OP) images.

It was observed that T1W–T2W Dixon OP images were insufficient to show the presence of erosion, but T1W–T2W Dixon FO and IP images were similar to those of the standard protocol (Figure 2).

**Figure 4:**
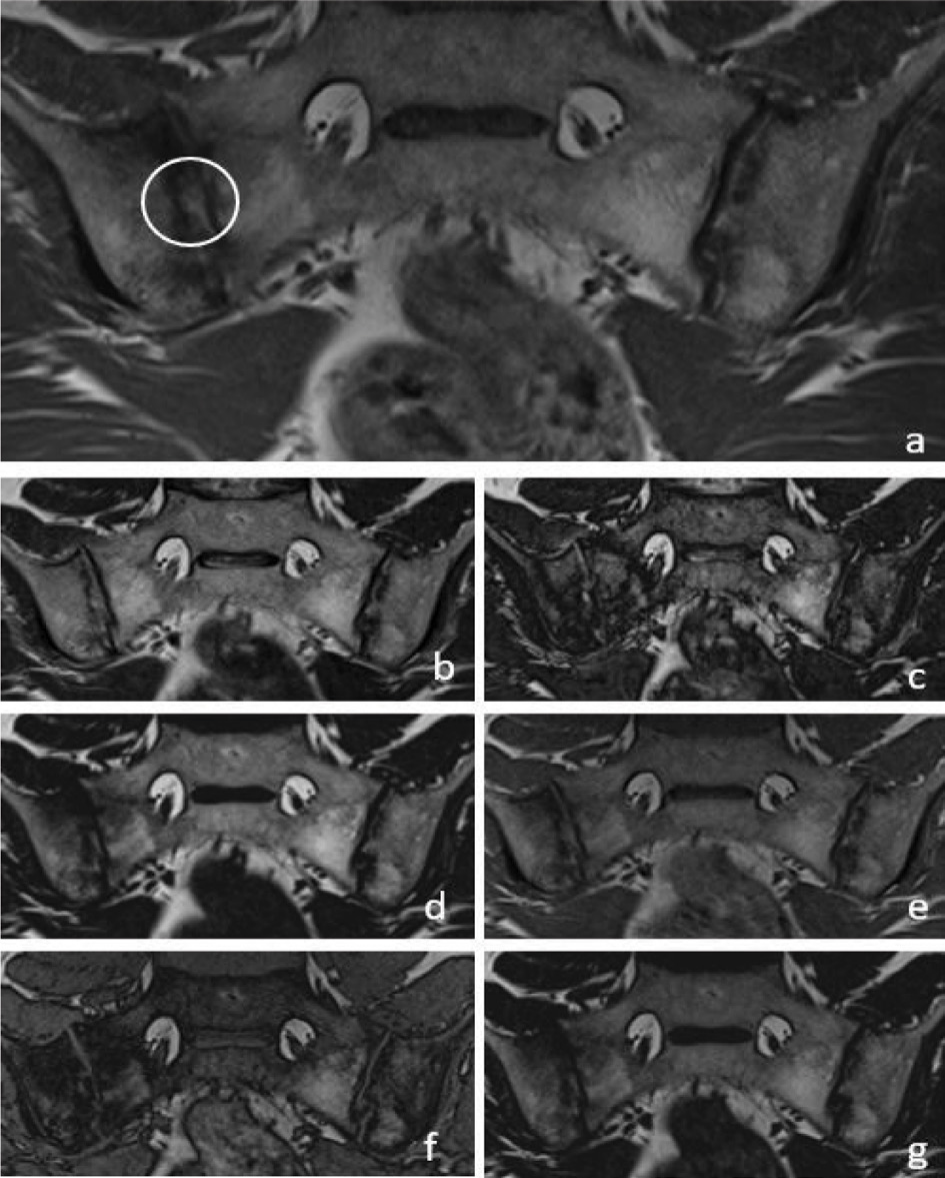
A 36‑year‑old male patient with backfill (circle) in the right sacroiliac joint on a T1W images **(a)**. T2W Dixon IP **(b)**, T2W Dixon OP **(c)**, T2W Dixon FO **(d)**, T1W Dixon IP **(e)**, T1W Dixon OP **(f)** and T1W Dixon FO **(g)** images are also shown in the figure.

T1W–T2W Dixon FO, IP and OP images were found to be inadequate compared with those of the standard protocol for revealing the presence of subchondral sclerosis (Figure 2). T1W Dixon OP images were found to be insufficient to show backfill (*P* = 0.016), and other Dixon images were found to be similar to the standard protocol (Figure 4).

### Quantitative assessments

The SNRs–CNRs for BME were calculated for 46 SIJ. The SNRs–CNRs of T2W Dixon WO images (mean SNR 79.64, mean CNR 45.1) were significantly greater than those of FS‑T2W images (mean SNR 34.9; mean CNR 20.2; *P* = 0.00 and *P* = 0.02, respectively).

The SNRs–CNRs for PFD were calculated for 23 SIJ. The SNRs–CNRs were significantly greater in T2W Dixon FO images than the standard protocol (*P* = 0.00, *P* = 0.00). Moreover, the SNRs–CNRs for PFD did not significantly differ among the T2W Dixon IP (*P* = 0.18, *P* = 1.00), T2W Dixon OP (*P* = 0.12, *P* = 1.00) and T1W Dixon FO (*P* = 0.054, *P* = 1.00) images and the standard protocol, but those obtained from T1W Dixon IP (*P* = 0.00, *P* = 0.002) and OP (*P* = 0.00, *P* = 0.04) images were significantly lower than those obtained using the standard protocol.

### Interobserver agreement assessment

Interobserver agreement regarding the presence of BME was almost perfect for the standard protocol and for T2W Dixon WO images. For erosion, the T2W Dixon kappa (*κ*) value was substantial, and the *κ* values for T1W Dixon and for the standard protocol were almost perfect. For subchondral sclerosis, the standard protocol *κ* value was substantial, and the *κ* values for T1W Dixon and for T2W Dixon were moderate. In the assessment of PFD, the T1W Dixon *κ* value was substantial, and the *κ* values for T2W Dixon and for the standard protocol were almost perfect. For backfill, the standard protocol *κ* value was moderate, and the T1W Dixon and T2W Dixon *κ* values were substantial. In the evaluation of ankylosis, the agreement for T2W Dixon was perfect, and the *κ* values were almost perfect for T1W Dixon and the standard protocol. The associated *κ* and *P* values are summarized in Table 4.

**Table 4:** Interobserver agreement assessment values at 214 SIJs for signs of active and chronic sacroiliitis.

	*K*	*P*
BME, T2W‑FS	0.93	0.01
BME, T2W Dixon	0.91	0.01
Erosion, T1W	0.84	0.01
Sclerosis, T1W	0.72	0.01
PFD, T1W	0.91	0.01
Backfill, T1W	0.53	0.01
Ankylosis, T1W	0.89	0.01
Erosion, T1W Dixon	0.81	0.01
Sclerosis, T1W Dixon	0.57	0.01
PFD, T1W Dixon	0.79	0.01
Backfill, T1W Dixon	0.66	0.01
Ankylosis, T1W Dixon	0.89	0.01
Erosion, T2W Dixon	0.78	0.01
Sclerosis, T2W Dixon	0.50	0.01
PFD, T2W Dixon	0.84	0.01
Backfill, T2W Dixon	0.80	0.01
Ankylosis, T2W Dixon	1.0	0.01

Diagnostic performance of T1W Dixon and T2W Dixon images.

The diagnostic performance of T1W–T2W Dixon images was calculated according to the standard protocol, which is accepted as the gold standard, and the details are presented in Table 5.

**Table 5:** The diagnostic performance of T1W and T2W Dixon sequences compared with the standard MRI protocol. PPV, positive predictive value; NPV, negative predictive value.

	MRI FINDINGS	SENSITIVITY (%)	SPECIFICITY (%)	PPV (%)	NPV (%)	ACCURACY (%)
T1W Dixon	Erosion	91.95	96.85	95.24	94.62	94.86
	Subchondral sclerosis	44.44	98.22	89.96	86.91	86.92
	PFD	86.96	99.48	95.24	98.45	98.13
	Backfill	57.14	100.00	100.00	98.57	98.57
	Ankylosis	80.00	99.52	80.0	99.52	99.07
T2W Dixon	BME	98.25	99.36	98.25	99.36	99.07
	Erosion	88.89	99.19	98.77	92.48	94.86
	Subchondral sclerosis	37.78	97.63	80.95	85.49	85.05
	PFD	86.96	98.95	90.91	98.44	97.66
	Backfill	57.14	100.00	100.00	98.57	98.57
	Ankylosis	80.00	100.00	100.00	99.52	99.53

## Discussion

In our study of images taken from 107 patients, we showed that T2W Dixon imaging is qualitatively sufficient to demonstrate BME and that its specificity and sensitivity are high enough for diagnostic performance analysis (Table 5). According to our quantitative evaluation, the SNRs–CNRs were found to be greater in T2W Dixon WO images than for the standard protocol. Therefore, BME is brighter and easier to distinguish from the background on T2W Dixon WO images. In addition, compared with those of the standard protocol, the diagnostic performance of T1W–T2W Dixon imaging for erosion, PFD and ankylosis was high enough (Table 5). However, the diagnostic performance of all T1W–T2W Dixon images was found to be low in demonstrating subchondral sclerosis. Additionally, the diagnostic performance in showing backfill is sufficient (Table 5). T1W Dixon OP images were found to be inadequate and other T1W–T2W Dixon images are adequate in demonstrating backfill in correlation tests.

There are few studies in literature comparing the T2W Dixon sequence with the standard protocol for the diagnosis of sacroiliitis. In the first study, which was conducted in 49 patients [7], no significant difference was found between T2W Dixon and the standard protocol for detecting BME. In this study, the quantitative evaluation of BME, CNRs was found to be greater for T2W Dixon WO images. In another study by Huang et al. with 107 patients [8], there was no significant difference between T2W Dixon WO images and the standard protocol for diagnosing BME, while the SNRs–CNRs were found to be significantly greater for T2W Dixon WO images. Another recent study that included 37 patients featured a comparison of T2W Dixon sequences with standard contrast‑enhanced imaging and showed that T2W Dixon WO images were quantitatively superior to fat‑suppressed T2W and contrast‑enhanced images in the diagnosis of BME [9]. Specifically, in literature, the T2W Dixon sequence was shown to be qualitatively similar to the standard protocol and quantitatively superior to the standard protocol for detecting BME, similar to our study [7–9].

There are studies in the literature with different results regarding the detection of chronic sacroiliitis findings with T2W Dixon imaging. In one study [7], while T2W Dixon FO and IP images showed similar results to those of the standard protocol, OP images were insufficient for revealing sclerosis. In contrast to that study, in our study, all T2W Dixon images were found to be inadequate for revealing sclerosis compared with the standard protocol (Table 3). In that same study [7], T2W Dixon OP and FO images were superior to the standard protocol and T2W Dixon IP images for demonstrating PFD. However, in our study, no significant difference was found between the T2W Dixon images and the standard protocol for demonstrating PFD (Table 3). In the quantitative evaluation of PFD, T2W Dixon OP images were found to be superior to the standard protocol in that study, whereas T2W Dixon FO images were superior in our study. Huang et al. [8] showed that the T2W Dixon sequence was superior to interobserver agreement in terms of erosion, backfill, PFD and ankylosis. In our study, the agreement was found to be high for the T2W Dixon sequence and the standard protocol for erosion, ankylosis and PFD but low for the T2W Dixon sequence in the detection of backfill (Table 5). In that same study [8], the SNRs–CNRs for PFD were determined on T2W Dixon FO images and T1W images, and similar to our study, the values were found to be greater for T2W Dixon FO images.

Athira et al. [9] revealed that T2W Dixon OP images were insufficient in detecting erosion, while FO and IP images were sufficient, similar to our results (Table 3). In this study, all images were similar in detecting the presence of PFD. However, in quantitative analysis, T2W Dixon FO images were superior in our study, while T2W Dixon OP images demonstrated superiority in this study. While all T2W Dixon images were insufficient for detecting sclerosis in our study (Table 3), T2W Dixon IP images were similar to the standard protocol for detecting sclerosis, but OP and FO images were insufficient in that study.

In a study [10] that compared the T2W Dixon sequence and the standard MRI protocol for detecting erosion in 23 patients with chronic sacroiliitis, T2W Dixon OP images were insufficient to show erosion, and the IP and FO images were similarly accurate to those of the standard protocol, which was similar to the results of our study as a demonstrated in Table 3.

T2W Dixon images demonstrate comparable results with the standard protocol in detecting erosion, ankylosis and PFD when evaluating our study alongside others in literature. However, there are notable discrepancies in the detection of sclerosis and backfill.

In our study, the interobserver agreement was almost perfect for the standard T2W‑FS and T2W Dixon images for the detection of BME. Interobserver agreement was found to be high for detecting erosion, PFD and ankylosis in the standard protocol and T1W and T2W Dixon images. However, interobserver agreement in detecting sclerosis and backfill was generally low in both the standard protocol and all Dixon images (Table 4).

In our study, T1W and FS‑T2W images were evaluated in the standard protocol, and the total imaging time of these sequences at our centre was 4 min and 25 s (2 min 44 s and 1 min 41 s, respectively; Table 1). The imaging time of T2W Dixon, which was shown in our analysis to replace the entire standard protocol in the diagnosis of active and chronic sacroiliitis, was 3 min and 11 s. By using only the T2W Dixon sequence, the imaging time is reduced, which will result in increased patient comfort for those presenting with low back pain; in addition, movement artifacts secondary to involuntary movements and the cost of imaging will decrease. According to our qualitative evaluations, the T1W Dixon sequence, which enables evaluation solely of chronic sacroiliitis signs, requires 2 min and 11 s and can substitute for the only T1W sequence in the standard protocol, which takes 2 min and 44 s (Table 1). In this way, the acquisition time will be partially reduced, but this is not a tremendous time gain. Considering all the qualitative and quantitative evaluations, we do not recommend the use of the T1W Dixon sequence in routine practice, since standard T1W images provide similar or superior results to T1W Dixon images.

Our study has several limitations. First, our study was a single‑centre study. Second, clinical‑laboratory and follow‑up findings were not included. Third, the number of patients with ankylosis and backfill was low (Table 3).

## Conclusions

This study revealed that the T2W Dixon sequence can be used alone for standard SIJ imaging, thus reducing acquisition time and cost and increasing patient comfort. We do not recommend the routine use of the T1‑weighted Dixon sequence in SIJ imaging, as it only allows for the evaluation of chronic sacroiliitis findings (such as subchondral sclerosis, PFD, ankylosis and filling), without significant advantages over the standard T1W sequences.
